# The Global Relationship Between the Prevalence of Diabetes Mellitus and Incidence of Tuberculosis: 2000-2012

**DOI:** 10.5539/gjhs.v7n2p183

**Published:** 2014-10-28

**Authors:** Alaa Badawi, Suzan Sayegh, Mohamed Sallam, Eman Sadoun, Mohamed Al-Thani, Muhammad W. Alam, Paul Arora

**Affiliations:** 1 Office of Biotechnology, Genomics and Population Health, Public Health Agency of Canada, Toronto, ON, Canada; 2 Public Health Department, Supreme Council of Health, Doha, Qatar; 3 Clinical Research Division, Supreme Council of Health, Doha, Qatar; 4 Public Health and Safety Department, Dubai Health Authority, Dubai, United Arab Emirates; 5 Division of Epidemiology, Dalla Lana School of Public Health, University of Toronto, Toronto, ON, Canada

**Keywords:** diabetes mellitus, tuberculosis, disease burden, global association

## Abstract

**Background:**

The dual burden of tuberculosis (TB) and diabetes mellitus (DM) has increased over the past decade with DM prevalence increasing in countries already afflicted with a high burden of TB. The coexistence of the two conditions presents a serious threat to global public health.

**Objective:**

The present study examines the global relationship between the prevalence of DM and the incidence of TB to evaluate their coexistence worldwide and their contribution to one another.

**Methods:**

This is an ecological longitudinal study covering the period between years 2000 to 2012. We utilized data from the WHO and World Bank sources and International Diabetes Federation to estimate prevalence of DM (%) and the incidence of TB (per 100,000). Measures of central tendency and dispersion as well as the harmonic mean and linear regression were used for different WHO regions. The association between DM prevalence and TB incidence was examined by quartile of DM prevalence.

**Results:**

The worldwide average (±S.D.) prevalence of DM within the study period was 6.6±3.8% whereas TB incidence was 135.0±190.5 per 100,000. DM prevalence was highest in the Eastern Mediterranean (8.3±4.1) and West Pacific (8.2±5.6) regions and lowest in the Africa (3.5±2.6). TB incidence was highest in Africa (313.1±275.9 per 100,000) and South-East Asia (216.7±124.9) and lowest in the European (46.5±68.6) and American (47.2±52.9) regions. Only countries with high DM prevalence (>7.6%) showed a significant positive association with TB incidence (*r*=0.17, *p*=0.013).

**Conclusion:**

A positive association between DM and TB may exist in some – but not all – world regions, a dual burden that necessitates identifying the nature of this coexistence to assist in developing public health approaches that curb their rising burden.

## 1. Introduction

An association between tuberculosis (TB) and diabetes mellitus (DM) has been recognized since the Roman era where it was indicated that DM increases the individual’s susceptibility to TB infection (Cooper et al., 1951; Ferrara, 1952; Brock, 1957; Dixon, 2007). Presently, more than 9.4 million people worldwide are affected by TB and over 1.7 million die from the disease every year (WHO, 2010). It is also estimated that one-third of the world population is infected with latent TB, where individuals are at risk of developing the active disease, particularly those with an impaired or compromised immune system (Corbett et al., 2003). These high rates of disease incidence render TB as a major public health problem in many developing low- to middle-income countries and regions such as those in Africa and Asia. Developing countries also experience a relatively elevated DM prevalence and are thought to have their highest DM rates by 2030 (Wild et al., 2004). Indeed, DM is another major public health problem where approximately six people die every minute from the disease worldwide (WHO, 2006). According to recent estimates by the International Diabetes Federation (IDF), the number of people currently living with DM globally is about 382 million. It is expected that DM will soon be the most prevalent health issue worldwide as the number of patients is expected to reach 592 million by 2035 (IDF, 2013). This increase in disease prevalence may be related to the growing adoption of various adverse environmental and behavioral risk factors as well as the longevity of patients with DM-related conditions, e.g., obesity, cardiometabolic disease and insulin resistance, secondary to improved healthcare settings (WHO, 2006; IDF, 2013).

The growing prevalence of DM poses a challenge for TB control as uncontrolled DM leads to a greater risk of developing TB. It has been noted that countries with an increased rate of DM prevalence also had a significant increase in the TB incidence (Goldhaber-Fiebert et al., 2011). In agreement, in both developed countries (Jeon & Murray, 2008) and developing regions such as Africa (Goldhaber-Fiebert et al., 2011), people with DM are at about threefold higher risk of developing TB compared to their healthy counterparts (Ottmani et al., 2010; Maurice, 2011). Furthermore, the autoimmune type 1 DM is similarly associated with TB susceptibility (Webb et al., 2009) as with the insulin-resistant type 2 DM. The latter comprises about 90% of the global cases of DM and a large proportion of those affected by both TB and DM burden (Gauld & Lyall, 1974; Chen et al., 2011). It is known that both type 1 and type 2 DM are linked to hyperglycemia (Brownlee, 2001) that impact the host defense mechanism and subsequently influence the HIV/AIDS-related TB (Gupta et al., 2011; Gray, & Coh, 2013; Martinez, & Kornfeld, 2014; Dooley, & Chaisson, 2009). Although the precise mechanism of this finding, however, remains to be fully understood, it is proposed that phagocytosis and bactericidal activity of neutrophils are impaired in DM patients (Martinez & Kornfeld, 2014). This is related to the reduced host defense to infection with extracellular bacteria. Host defense to mycobacterial infection is largely mediated by cellular immunity and related cytokines, such as IFN-γ and IL-12. Serum level of these cytokines are reduced in TB patients with DM, and this is thought to be involved in the high incidence of TB in subjects with DM (Martinez & Kornfeld, 2014; Dooley, & Chaisson, 2009).

In general, the dual burden of TB and DM has increased dramatically over the past decade with DM prevalence increasing in countries already afflicted with a high burden of TB. The confluence of the two conditions presents a serious threat to global public health. Opportunities to further understand the global link between the two diseases may permit developing public health-related polices and actions to curb their rising burden. The present study was undertaken in an attempt to examine the global relationship between the prevalence of DM and incidence of TB in order to evaluate their coexistence in the different world regions and characterize the nature and extent of their association.

## 2. Materials and Methods

### 2.1 Source of Data

In order to understand the effect of DM on TB globally, we examined the link between the prevalence of DM and the incidence of TB globally between 2000 and 2012. We conducted a longitudinal ecological study covering the study period and used a range of secondary data sources available in the public domain (see below). Estimates from 196 countries were divided into the WHO six regions, i.e. African (AFR), American (AMR), Eastern Mediterranean (EMR), European (EUR), South-East Asia (SEA), and Western Pacific (WP) Regions.

#### 2.1.1 TB Incidence

Disease incidence in general is defined as the number of new cases during some period of time; one year in the present study (Dorland’s Medical Dictionary, 2012). TB incidence gives an indication of the burden of TB in a population as well as the size of the task faced by a national TB control program. The estimated number of new and relapse TB cases arising within the study period is expressed as the rate per 100,000 population. Data were obtained from the WHO global tuberculosis control report (WHO, 2013a). All forms of TB were included such as cases in people living with HIV. In this report, TB estimates are based on annual case notifications, assessments of the quality and coverage of TB notification data, national surveys of the prevalence of TB disease and on information from death (vital) registration systems. Estimates of incidence for each country are derived using one or more of the following approaches, depending on the available data: (a) case notifications/estimated proportion of cases detected; (b) prevalence/duration of condition; or (c) deaths/proportion of incident cases that die. Estimates are also produced at global level, for WHO regions and for World Bank Income Groups as indicated in the WHO global tuberculosis control report (WHO, 2013b). Routine surveillance data provide a proper estimates of incidence in countries where the majority of incident cases are treated and notified to the WHO. Where the proportion of cases notified is consistent over time, trend in incidence was judged from trends in notified cases (WHO, 2013a).

#### 2.1.2 DM Prevalence

Prevalence is the proportion of a population found to have the disease and it is derived by comparing the number of people found to have the condition with the total number of the studied population. It is usually expressed as a fraction; a percentage in the present study (Kenneth, 2012). We utilized the 2000–2012 editions of the International Diabetes Federation Diabetes Atlas (IDF, 2000; IDF, 2003; IDF, 2007; IDF, 2012) in addition to the data visualization tool of the IDF to generate the prevalence of DM from the same 196 countries and territories where the TB incidence rates were extracted for the same period of time. We used the comparative prevalence estimates in adults (20–79 years) that have been calculated by assuming that every country and region has the same age profile (the age profile of the world population has been used). This reduces the effect of age differences between countries and regions, and renders the figure ideal for comparisons. For example, the comparative prevalence for 2010 shows that Samoans (7.7%) are in fact more prone to have diabetes than are Japanese (5.0%). The comparative prevalence should not be used for assessing the proportion of people within a country or region that have DM.

### 2.2 Data Analysis

Data on estimated prevalence (DM) and incidence (TB) for individual countries were combined to give regional estimates of association. We carried out a time-trend to substantiate the dynamics of the TB incidence and DM prevalence in the different world regions over the study period. Pattern of change in the rates of the two diseases was examined using the linear regression analysis. Descriptive analysis and harmonic mean were carried out to obtain the average of rates. Harmonic mean – compared to the arithmetic mean – tends to mitigate the impact of large outliers and aggravate the impact of small ones. We also developed a linear regression models to estimate the association between DM and TB within the different regions. In their analysis of causes of death, to determine the global burden of disease, Murray et al. (2010) used the Cause of Death Ensemble model strategy to develop ensembles of the best performing models that met two plausibility criteria. The first is that the direction of the regression coefficient for a covariate is in the expected direction. The second is that the coefficient has a *p* value of <0.05. In our analysis we only employed the second criteria of Murray et al. (2010). We utilized the linear regression approach taking into consideration the change of the prevalence (DM)/incidence (TB) rates within the study period. Furthermore, based on the hypothesis that DM prevalence influences TB incidence, we divided the extent of DM comparative prevalence into four quartiles. The relationship between the DM prevalence and TB incidence stratified by DM prevalence was further evaluated within each quartile. Data was analyzed using StatPlus for Mac, 2011, version 5.8; AnalystSoft Inc.

## 3. Results

**Figure 1:**
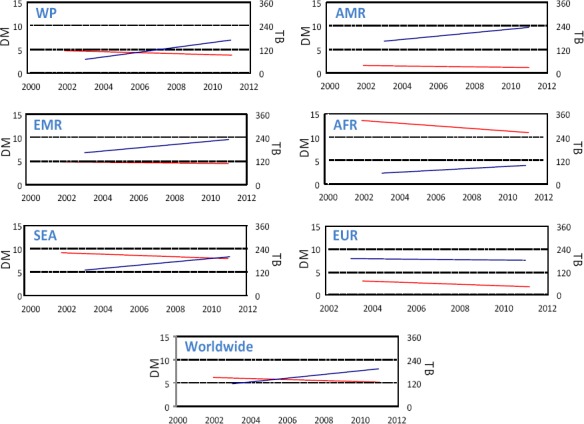
A comparative trend for the diabetes mellitus prevalence and tuberculosis incidence worldwide and across different WHO regions between years 2000 and 2012

The present study explores the relationship between TB and DM globally. As shown in Figure 1, there was an overall increase in the prevalence of DM with a decline in the incidence of TB within the study period of 2000 to 2012 both worldwide and in the various WHO regions. AFR region had the highest TB incidence whereas AMR and EUR regions had the lowest. The highest prevalence of DM, however, was seen in the EMR and AMR regions with the lowest trend rates in the AFR region. A non-significant inverse relationship between the two rates appeared in the different world regions (Table 1).

**Table 1:** The average prevalence of diabetes mellitus (DM) and incidence of tuberculosis (TB) worldwide and their time-trend between the years 2000 and 2012

Region^1^	DM comparative prevalence (%)		TB incidence (per 100,000)		Time-trend Analysis^2^		TB-DM Correlation (*r*)^3^

	Mean±SD	Harmonic Mean	Mean±SD	Harmonic Mean	TB	DM	
World	6.6 ± 3.8	4.2	135.0 ± 190.5	17.9	-0.01	**+0.21**	-0.36
AMR	7.7 ± 2.9	6.5	47.2 ± 52.9	14.0	-0.10	**+0.29**	-0.13
EMR	8.3 ± 4.1	6.3	86.4 ± 132.9	20.6	-0.13	**+0.37**	-0.39
AFR	3.5 ± 2.6	2.2	313.1 ± 275.9	138.1	-0.07	**+0.48**	-0.27
SEA	5.1 ± 2.4	3.8	216.7 ± 124.9	136.4	**-0.13**	**+0.32**	-0.23
EUR	7.3 ± 2.4	5.7	46.5 ± 68.6	9.0	-0.06	**+0.21**	-0.37
WP	8.2 ± 5.6	4.9	142.9 ± 141.5	32.1	-0.01	+0.16	-0.14

Note:

1 AFR, African Region; AMR, American Region; EMR, Eastern Mediterranean Region; EUR, European Region, SEA, South-East Asia Region and WP, Western Pacific Region.

2 Trend analysis was calculated by linear regression analysis. Values represent the regression coefficient. Values in bold are statistically significant at *p*<0.05. Negative and positive values represent a time-dependent decline and increase in the disease rates, respectively.

3 Correlation analysis was carried out between TB and DM across the entire study period. *r* represents the correlation coefficient. No significant association was found between the two conditions among the different world regions.

The average DM prevalence and TB incidence (±S.D.) over the study period is shown in Table 1. The worldwide average DM prevalence and TB incidence were 6.6±3.8% and 135.0±190.5 per 100,000, respectively. The highest rates of DM prevalence were in the EMR (8.3±4.1%) and WP (8.2±5.6%) regions and lowest in the AFR region (3.5±2.6%). On the other hand, the AFR region had the highest TB incidence (313.1±275.9 per 100,000) followed by the SEA (216.7±124.9) and EUR (46.5±68.6) and AMR (47.2±52.9) regions. Time-trend analysis has shown that there is an overall decline in TB incidence in the world over the study period and an increase in the prevalence of DM incidence. However, no significant negative relationship was obtained to implicate an inverse association between the two conditions during the studied period in the different world regions. Harmonic means deviated from the arithmetic means were – as expected –of a lesser average TB and DM rates.

*Note:* Trends were calculated by linear regression along the study period. The DM axis represents the prevalence (%) of diabetes mellitus (blue line) whereas the TB axis represents the incidence (per 100,000) of tuberculosis (red line). The different panels show the trends in the different WHO regions as well as worldwide. The different panels have the similar TB and DM scales for the purpose of inter-regional comparison. AFR, African Region; AMR, American Region; EMR, Eastern Mediterranean Region; EUR, European Region, SEA, South-East Asia Region and WP, Western Pacific Region. Results of time-trend analysis for TB and DM are shown in Table 1.

**Figure 2:**
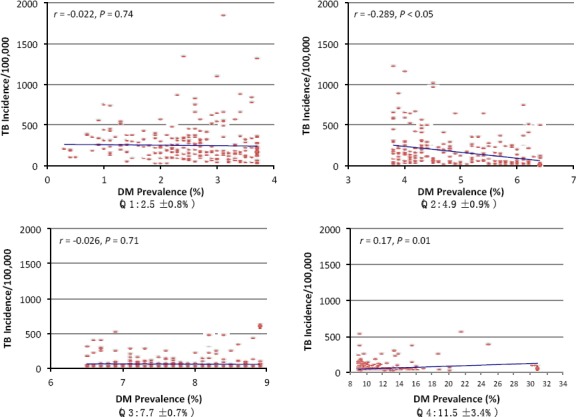
The association between diabetes mellitus and tuberculosis stratified by diabetes prevalence between years 2000 and 2012

*Note:* The extent of DM comparative prevalence was divided into four quartiles. The relationship between the DM prevalence (%) and TB incidence (per 100,000) stratified by DM prevalence was further evaluated within quartile. Numbers in parenthesis represent the mean±SD of DM prevalence of the corresponding quartile and within the study period. Extent of association was evaluated by linear regression analysis. The different panels have the similar TB scale for the purpose of inter-quartile comparison.

In an attempt to further understand the association between DM prevalence and TB incidence worldwide and at different world regions, we divided the DM prevalence into quartiles and examined the correlation between the two conditions within each quartile (Figure 2). No positive association between the two diseases was observed in the lowest three quartiles where the DM prevalence was at low to moderate rates, i.e., below 4.8%. Positive association between DM prevalence and TB incidence was only observed, however, in countries with high DM prevalence rates (>7.6%, *r*=0.17, *p*=0.013). The countries with a comparative DM prevalence over 7.6% were primarily in the WP, EMR, and SEA regions.

## 4. Discussion

An effective preventive strategy for TB has been the concern of the health authorities and research worldwide for decades. Undeniably, the current protocols against the spread of TB have reduced its incidence as it shows in its declining rates worldwide and in different world regions (Figure 1). However, the TB rates are still high. Focus has been directed over the past decade on some of the previously untargeted risk factors involved in the spread of TB, particularly, increasing DM prevalence around the world. It is well known that DM impairs the immunity of patients (Badawi et al., 2010) leading to a higher baseline burdens with *Mycobacterium tuberculosis* (Dooley & Chaisson, 2009). In agreement, several studies have shown that DM patients are more susceptible to TB and suffer from relatively severe illness due to their immuno-compromised status (Dooley & Chaisson, 2009). The impact of an increasingly globalized world on disease burden goes beyond the infectious disease to affect chronic conditions as well. The latter are rising in prevalence in middle- and low-income countries as populations age and lifestyles and diets change. In 2000, developing countries were estimated to carry 67% of the global burden of DM, but this proportion is predicted to rise to 78% by 2030 (Wild et al., 2004). These rates were paralleled by an increased incidence of TB from ~4 million in 1990 to ~5 million in 2010 (WHO, 2010).

As shown by our results, DM increases with time in almost all world regions (Table 1) with the highest rates in the EMR and WP regions and lowest in the AFR area. The increased rate of DM in these areas is due to the change in lifestyle, which is mainly linked to poor nutrition and lack of physical inactivity (Hu, 2011). The EMR countries are characterized by the young population with a majority of Arabs. In Arab countries, 54% of the population is below 25 years of age (Mirkin, 2010). This is in line with other characteristics leading to increased exposure to diabetes risk factors such as obesity, physical inactivity, unhealthy diet, and hyperlipidemia (Musaiger & Al-Hazzaa, 2012; Badawi et al., 2012). Such conditions are also established in the WP region where DM prevalence is high due to the similar factors (Chan et al., 2013). This would lead to an elevated risk of DM among this population at a later stage in life and possibly a subsequent increased incidence of TB. Even though our results show that the AFR region has the lowest rates of DM, diabetes is escalating in this region, likely due to ageing, population growth, rapid urbanization, in addition to increased consumption of processed foods and reduced physical activity (Mbanya et al., 2010). TB rates were the highest amongst the AFR and SEA regions, perpahs due to the low socio-economic condition, lack of control programs and awareness among people to cope with the disease, as well as lack of proper nutrition and less than optimal immunity status in a large proportion of the population. Although TB rates decreased with time in the various world regions (Table 1), this change was not signifanct. Developed countries such as the AMR and EUR regions have shown the most pronounced decreased incidence of TB as a result of increased awareness and implementing community-based educational programs about the disease, provision of appropriate treatment and improvement of surveillance systems.

There existed a positive association between DM prevalence and TB incidence in countries with high DM prevalence rates (>7.6%, *r*=0.17, *p*=0.013). Although this association *per se* cannot imply causality and it is somehow weak, it may, however, provide some evidence suggesting that introducing effective strategies for DM prevention may reduce TB incidence in the low- to middle income countries. Most of these countries were in the WP, EMR and SEA regions. Accordingly, if TB and DM are casually associated, TB control strategies can be targeted at the poorest populations that are at risk, to address a major determinant of disease – specifically, DM. Although a weak – albeit significant – direct association was observed in some regions between TB and DM (and may not suggest causality), a more successful strategy can be proposed to be directed towards circumventing the risk factor of DM itself, i.e., preventive strategies for “at-risk” subpopulations. It is expected that, with the escalating rates of DM and obesity around the world, in addition to the persistent high levels of TB in middle- and low- income countries, the number of individuals having both diseases will further increase within the coming decades (Dooley & Chaisson, 2009). Indeed, type 1 and type 2 DM share a similar array of complications that develop as a consequence of the DM characteristic hyperglycemia (Brownlee, 2001). A recent study (Gupta et al., 2011) demonstrated that DM increases the risk for pulmonary TB but not the extrapulmonary disease in contrasts to the TB related to HIV/AIDS (mainly extrapulmonary) (Gray & Coh, 2013), suggesting that the host defense mechanisms impacted by DM may be particularly relevant to TB (Martinez & Kornfeld, 2014). Furthermore, hyperglycemia and cellular insulinopenia, influence macrophage and lymphocyte functions which, subsequently, lead to a status of reduced immune response (Dooley & Chaisson, 2009).

We observed a non-significant inverse relationship between the TB incidence and DM appeared in the AFR, SEA, and WP regions which may reflect the lack of significant direct association between the two conditions as noted in the three quartiles where DM prevalence was at low to moderate rates, i.e., below 4.8%. Additionally, a significant negative correlation between DM and TB was shown in the second quartile of DM prevalence (*r*=-0.289, *p*<0.05). Countries within this quartile are mostly from the AMR and EUR regions. These areas experience decreased rates of TB during the study period as a result of increased awareness accompanied by appropriate treatment and improvement of surveillance systems while still adopting practices that increase the incidence of DM, *e.g*., sedentary life-style and elevated consumption of dietary fat.

Although preventive strategies for TB and DM are available for the low- and middle- income countries, the limited resources hinder the generation of associated public health measures. The lack of political commitment for the concept of disease prevention together with the shortage in funding and the scarcity of human resources in addition to the diverse needs in public health policies and actions in the developing world, all culminate into less effective strategies in combating TB and DM (Miranda et al., 2008; Epping-Jordan et al., 2005; Borgdorff et al., 2002). In addition to public health policies, these countries require a stronger healthcare system that provides comprehensive, accessible, community-based, healthcare programs – including preventive, curative and rehabilitative care–for both communicable and non-communicable diseases (Miranda et al., 2008; Epping-Jordan et al., 2005).

The present study has several limitations since it was assembled using available open data source. Some countries with a less-than-optimal DM surveillance system or a proper community-based screening might have underestimated DM prevalence. This fact may levy some limitations on the use of the IDF data and render it as a just a guide to further generate more accurate prevalence estimates to be able to develop national or international intervention strategies. Furthermore, although a direct correlation between the two diseases was observed primarily in less-developed regions of the world, it was a weak overall association and – given the nature of the data – may not implicate causality between the TB and DM. Given this limitation, our hypothesis stipulating that DM prevalence in a given country/region influences TB incidence is only thought to take place in some regions of the world. The majority of the world regions, however, exhibited an inverse association between the two diseases, an observation that warrants further studies to identify the nature and extent of the coexistence between the two conditions.

In conclusion, the dual burden of TB and DM raises a public health concern where a refined understanding is needed with respect to TB susceptibility in diabetic individuals. Furthermore, TB and DM are relatively common, both in low-income and high-income countries (Stevenson et al., 2007; Dooley et al., 2009; Restrepo et al., 2007). This situation may impose a serious burden to public health services by way of managing the costs of communicable and non-communicable diseases. Public health policies and actions may be tailored to target increased prevalence of DM as a potential effort towards curbing high TB incidence, particularly in regions where the rates of both conditions are rising.
